# Perceived STEAM-integrated music education and students’ innovative competence: parallel indirect associations through creative self-efficacy and context-specific learning engagement

**DOI:** 10.3389/fpsyg.2026.1923767

**Published:** 2026-08-28

**Authors:** Jianyun Wei, Yun Li, Weiyi Huo

**Affiliations:** 1 Department of Global Convergence, Kangwon National University, Chuncheon, Republic of Korea; 2 Art College, Shandong Management University, Jinan, China

**Keywords:** bootstrap indirect association, confirmatory factor analysis, creative self-efficacy, innovative competence, learning engagement, measurement invariance, sensitivity analysis, STEAM-integrated music education

## Abstract

Innovative competence is an important educational outcome, but evidence from music-centered STEAM settings remains limited. This study examined whether students’ perceptions of STEAM-integrated music education (SIM-E) were associated with innovative competence (IC) and whether creative self-efficacy (CSE) and context-specific learning engagement (LE) accounted for distinct portions of that association. A cross-sectional survey was completed by 427 middle school and university students in Shandong Province, China. Maximum-likelihood confirmatory factor analysis supported a four-factor measurement model, *χ*^2^(428) = 489.87, CFI = 0.986, TLI = 0.985, RMSEA = 0.018, and SRMR = 0.038; the one-factor model fit substantially worse (CFI = 0.601, RMSEA = 0.099). HTMT values ranged from 0.319 to 0.533. Multigroup CFA further supported configural, metric, and scalar measurement invariance across middle-school and university participants (CFI = 0.975–0.977; absolute ΔCFI ≤ 0.002). In standardized composite-score regressions controlling for gender, age, prior music-learning experience, and prior STEAM experience, the adjusted total SIM-E–IC association was *β* = 0.331 (*p* < 0.001), and the residual direct association after CSE and LE was *β* = 0.194 (*p* < 0.001). With 5,000 bootstrap resamples, the specific indirect estimates through CSE (0.067, 95% CI [0.028, 0.111]) and LE (0.070, 95% CI [0.038, 0.106]) both excluded zero. Removing LE1, LE5, LE8, and LE9 yielded a reduced five-item engagement composite (LE-R); the LE-R indirect estimate remained 0.067 (95% CI [0.035, 0.103]), and the paired change from the full-LE estimate was 0.003 (95% CI [−0.010, 0.016]). An expanded-control sensitivity model that additionally adjusted for music major, digital-music-tool use, and school type also retained both specific indirect associations. Stage-specific bootstrap differences between middle-school and university estimates included zero. An exploratory ordered SIM-E → CSE → LE → IC product was also nonzero (0.040, 95% CI [0.024, 0.060]), but the cross-sectional design cannot establish temporal sequencing. Composite reliability was acceptable and the four-factor structure and discriminant validity were supported, whereas convergent-validity evidence remained mixed because AVE was below 0.50 for SIM-E, LE, and IC. The findings are therefore interpreted as robust parallel cross-sectional indirect associations rather than as causal or temporally ordered mediation.

## 1 Introduction

Innovative competence—the capacity to generate, develop, and apply novel and useful ideas—is increasingly treated as an educational outcome across school and university settings. STEAM education addresses this goal by connecting science, technology, engineering, the arts, and mathematics through inquiry, design, and authentic problem solving. Yet STEAM is not a single, uniform intervention: definitions, disciplinary configurations, and implementation practices vary substantially across studies (Perignat and Katz-Buonincontro, 2019).

Aguilera and Ortiz-Revilla (2021) synthesized research comparing STEM and STEAM in relation to student creativity and characterized the evidence base as heterogeneous rather than as a meta-analytic estimate of a single effect. Perignat and Katz-Buonincontro (2019) likewise documented wide variation in how STEAM is defined and enacted. Empirical studies reinforce this implementation-sensitive view: Conradty and Bogner (2019) found that a brief STEAM module supported knowledge and motivation but did not alter all self-reported creativity indicators, whereas Conradty et al. (2020) reported links among participation, self-efficacy, and creativity-related cognition across several modules.

Interdisciplinary curriculum reform has encouraged schools to combine music with inquiry, design, and digital production. Such integration varies widely, and the arts can be reduced to presentation rather than used as a mode of inquiry. Here, music-centered STEAM means that a musical question, process, product, or standard of quality organizes the task. Inquiry, collaboration, technology use, and revision are treated as general STEAM practices enacted through that musical focus, not as features unique to music.

Music can serve as a contextually specific carrier for interdisciplinary learning. In a SIM-E lesson, students might examine acoustic principles while constructing instruments, use digital tools to manipulate sound, apply mathematical structures to rhythm or harmony, and make esthetic decisions during composition or performance. Embodied action, temporal patterning, collaboration, and immediate auditory feedback give these projects a coherent musical focus while remaining consistent with broader STEAM principles (Liao, 2016; Quigley et al., 2017).

The study does not compare music-centered STEAM with other STEAM models. It therefore addresses a contextual rather than a comparative question: whether perceived SIM-E is associated with IC and whether CSE and LE account for distinguishable portions of that association within a music-organized setting. Improvisation, embodied activity, esthetic judgment, and auditory feedback characterize that setting, but they were not measured as separate mechanisms.

### 1.1 Music as a STEAM carrier and the need for mechanism-oriented evidence

**Figure 1:**
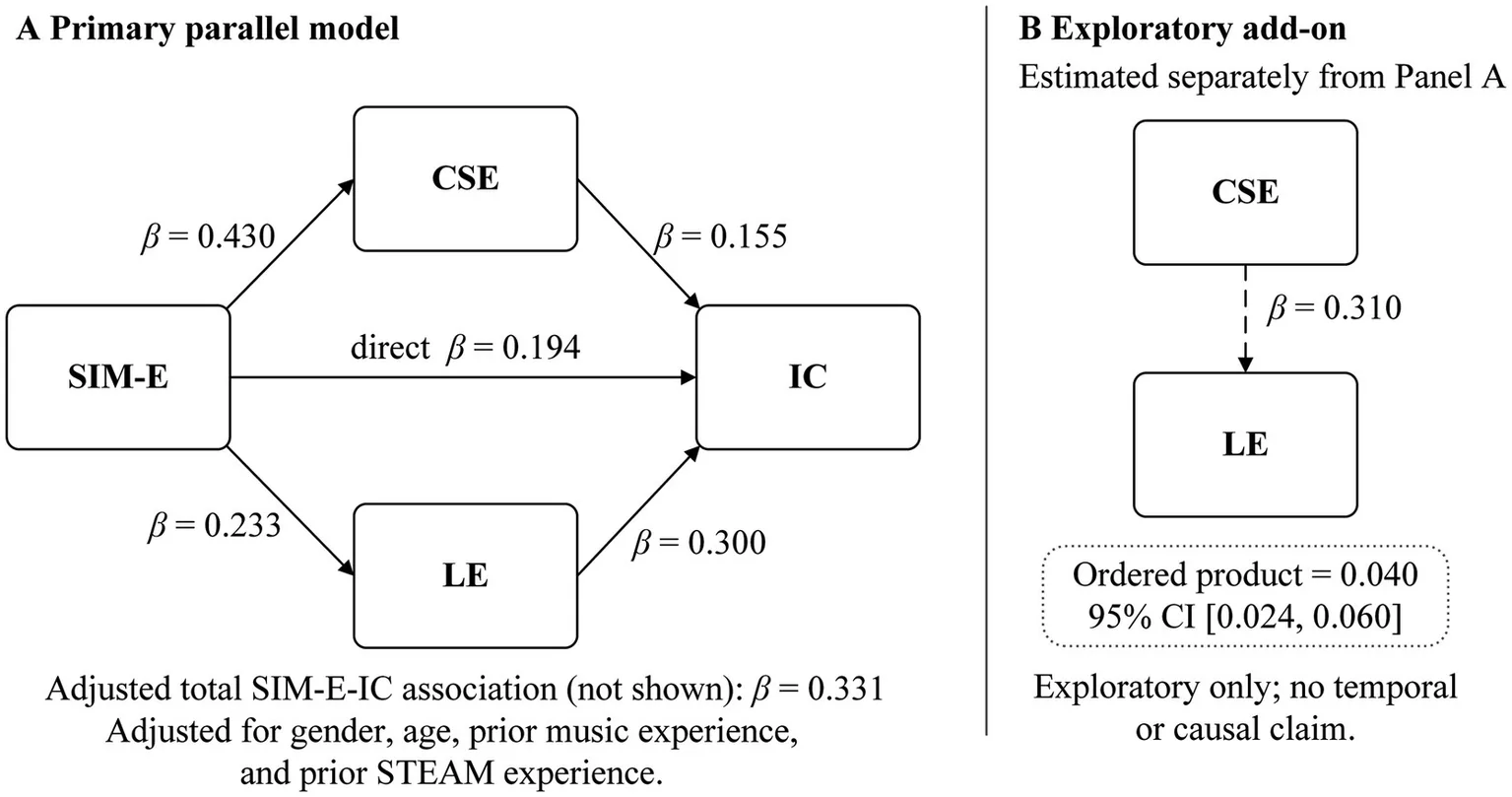
Observed composite-score path models regenerated from the final coefficients reported in Table 11. Panel **(A)** shows only the primary parallel model adjusted for gender, age, prior music experience, and prior STEAM experience; all primary paths are solid and labeled with standardized coefficients. Panel **(B)** isolates the additional CSE → LE coefficient from the separately estimated exploratory ordered model; the dashed arrow and separate panel deliberately reduce its visual prominence. The adjusted total SIM-E–IC association, not drawn, was *β* = 0.331. The specific indirect estimates and bootstrap intervals are reported in Table 12 and Figure 2. The exploratory ordered product was 0.040 (95% CI [0.024, 0.060]) and is not interpreted as evidence of temporal sequencing.

**Figure 2:**
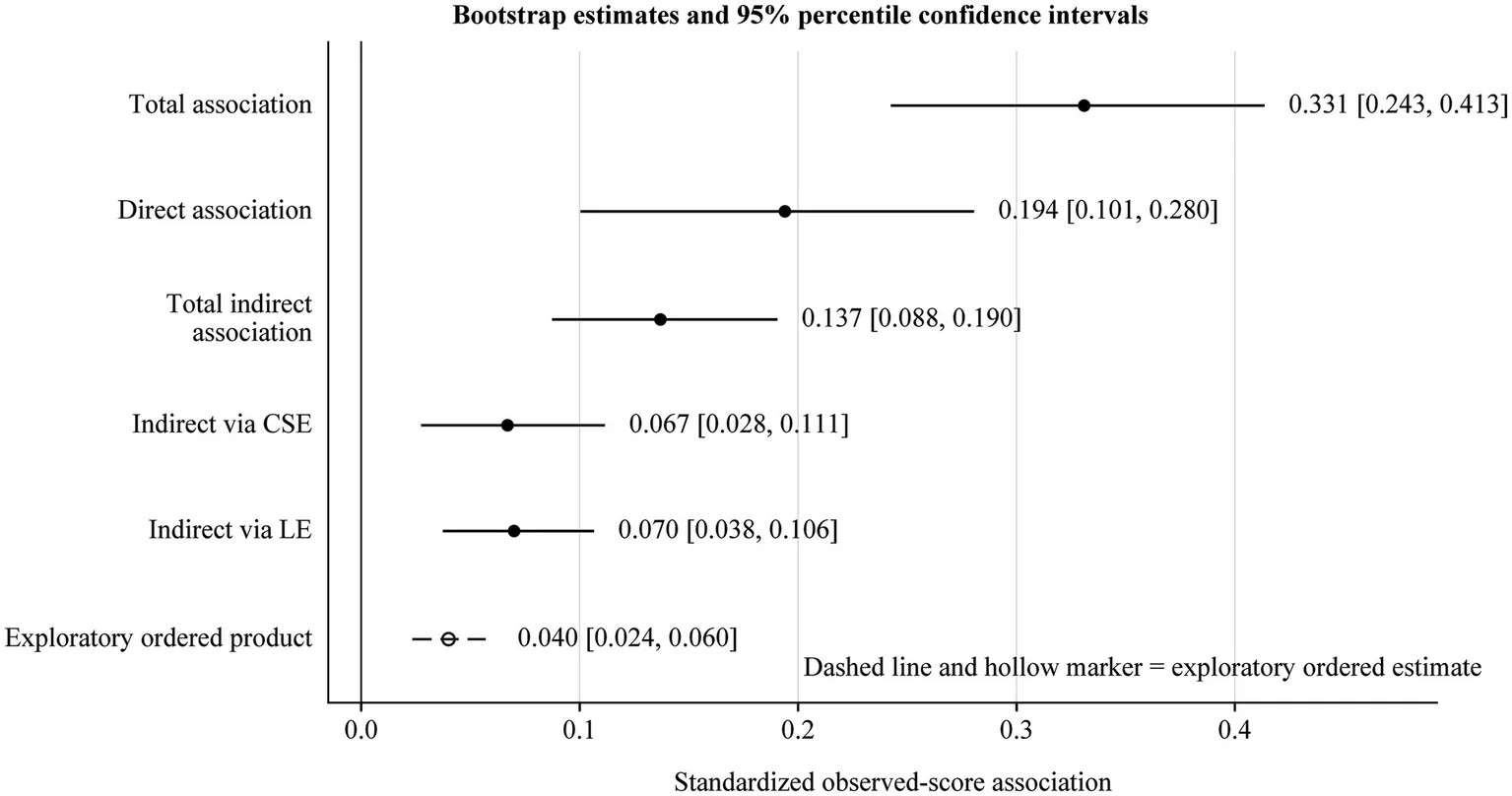
Bootstrap estimates and 95% percentile confidence intervals from 5,000 resamples, regenerated from the final values reported in Table 12. Filled markers and solid intervals denote estimates from the primary parallel observed-score model; the hollow marker and dashed interval denote the exploratory ordered product. The LE-specific indirect estimate was 0.070 (95% CI [0.038, 0.106]), and the exploratory ordered estimate was 0.040 (95% CI [0.024, 0.060]). The reduced-item LE-R sensitivity estimate was 0.067 (95% CI [0.035, 0.103]) and is reported in Table 8 rather than plotted.

Music learning combines auditory discrimination, temporal organization, bodily coordination, affective expression, symbolic representation, and social interaction. Performance, composition, improvisation, and digital sound design require learners to detect patterns, manipulate structures, negotiate esthetic choices, and respond to feedback. Experimental work has linked music-feedback exercise with divergent-thinking performance (Fritz et al., 2020), while neuroscience research identifies sustained musical practice as a useful model for experience-dependent plasticity (Schlaug, 2015). These findings motivate, but do not by themselves establish, music-specific effects within SIM-E (Figures 1, 2).

Within SIM-E, sound, image, gesture, notation, and digital interfaces can function as complementary representational resources. Instrument construction can make resonance and frequency tangible; composition and improvisation create open-ended problems; and ensemble work requires ideas to be tested, negotiated, and revised. Classroom studies of STEAM also emphasize authentic problems, disciplinary integration, collaboration, and metacognitive reflection as central features of students’ learning experiences (Bush et al., 2020; Herro et al., 2017).

These features characterize the learning context rather than additional variables in the model. The survey measured SIM-E, CSE, LE, and IC, but did not manipulate disciplinary components or assess music-specific cognitive processes. Accordingly, the analysis concerns associations within a music-organized STEAM setting and cannot isolate an effect attributable to music itself.

Research focused specifically on music-centered STEAM remains less developed than the broader STEAM literature. Existing reviews describe varied curriculum designs and inconsistent uses of the STEAM label (Kwan and Wong, 2021; Yim et al., 2025). Examining the proposed associations in a music-organized setting can therefore extend the contextual evidence base, although a non-music comparison would be required to identify any music-specific effect.

The study therefore asked whether perceived SIM-E was associated with IC and whether CSE and context-specific LE showed separate, parallel indirect associations with IC. A CSE → LE ordering was retained only as a secondary exploratory check because the cross-sectional design cannot establish temporal priority.

### 1.2 Theoretical foundation and research objectives

The model combines social cognitive theory with a multidimensional account of learning engagement. Social cognitive theory emphasizes reciprocal relations among environments, beliefs, and behavior, with self-efficacy shaping how learners approach challenge and persist (Bandura, 1997). Creative self-efficacy is the belief that one can produce creative outcomes. It has been associated with creative performance in workplace research (Tierney and Farmer, 2002), with creative beliefs among secondary students (Beghetto, 2006), and with antecedents and consequences across empirical studies reviewed by Puente-Díaz (2016).

Learning engagement includes behavioral participation, emotional involvement, and cognitive investment (Fredricks et al., 2004). Skinner et al. (2009) showed that engagement and disaffection can be differentiated through behavioral and emotional indicators, while Reeve and Tseng (2011) argued that agentic contribution is also relevant when students shape their learning activities. In interdisciplinary projects, these dimensions are reflected in sustained effort, response to feedback, collaboration, and revision.

The theories play complementary rather than sequential roles in the primary model. SIM-E represents the perceived learning environment, CSE a domain-relevant belief, LE an engaged learning state, and IC the outcome. Social cognitive theory motivates the CSE route, whereas engagement theory motivates the LE route. Because the data are cross-sectional, the primary framework treats CSE and LE as parallel correlates without assigning temporal priority. A CSE → LE ordering is examined only as an exploratory covariance pattern, not as a confirmed psychological sequence.

The model combines established social-cognitive and engagement perspectives rather than proposing a new general theory. Its contribution to general STEAM research is to examine efficacy beliefs and engagement as theoretically distinct but parallel correlates in the same model. Its contribution to music education is contextual: it examines those associations when musical problems, products, and evaluative criteria organize interdisciplinary work. The revised interpretation therefore prioritizes the parallel indirect associations and treats any ordered CSE → LE path as exploratory. Cross-sectional composite scores cannot establish causal mediation, temporal sequence, or effects unique to music.

## 2 Literature review

SIM-E is defined here as learning in which a musical problem or product provides the central purpose of an interdisciplinary task. The focal question, representational medium, intended product, and criteria for evaluation remain musical, while scientific, technological, engineering, or mathematical knowledge is used to understand, design, or improve the musical work. This definition follows transdisciplinary STEAM approaches that emphasize authentic problems and purposeful disciplinary integration (Liao, 2016; Quigley et al., 2017).

The nine SIM-E indicators therefore combine two conditions. First, each item is anchored to music learning, musical sounds or structures, musical expression, or a musical product. Second, the item requires inquiry, design, technology use, collaboration, or revision involving at least one additional STEAM discipline. The scale measures students’ perceived enactment of general STEAM principles in a music-organized setting; it does not measure a causal mechanism unique to music.

IC is defined as students’ perceived capacity to generate, develop, evaluate, and apply novel and useful ideas in learning and problem-solving contexts. The construct is broader than divergent thinking alone because it includes implementation and transfer. Runco and Acar (2012) clarify that divergent thinking is an indicator of creative potential rather than creativity itself. Using multilevel data from Chinese children and adolescents, Chen et al. (2024) likewise examined a broad innovation-related outcome influenced by individual and school-context factors.

SIM-E should be positively associated with IC because interdisciplinary music projects require learners to shift among concepts, tools, and representational systems. Designing a digital soundscape, for example, involves acoustic understanding, software decisions, compositional planning, esthetic evaluation, and revision after feedback. Repeated participation in such tasks provides practice in flexible problem representation and idea implementation. Given the study design, the expected relationship is stated as an association.

### 2.1 Indirect role of creative self-efficacy

Creative self-efficacy is a learner’s belief in the capacity to produce creative outcomes (Beghetto, 2006; Tierney and Farmer, 2002). Social cognitive theory identifies mastery experience, observation, social persuasion, and affective states as sources of efficacy beliefs (Bandura, 1997). Music-centered projects can provide these sources through iterative production, peer modeling, public feedback, and visible improvement. Large-sample STEAM research has also linked participation with self-efficacy and creativity-related cognition (Conradty et al., 2020).

CSE is expected to carry part of the SIM-E–IC association because students who believe they can produce creative work are more likely to propose ideas, take intellectual risks, and persist when projects become uncertain. In interdisciplinary music tasks, this confidence supports experimentation, revision, and the combination of digital and acoustic resources. The proposed indirect association is theoretically meaningful, but the cross-sectional design cannot establish a temporal mediating process.

This indirect route is also relevant for curriculum design. Adding technological or scientific content to music lessons is unlikely to be sufficient unless tasks allow visible creative progress, manageable challenge, and feedback that strengthens students’ agency as creators. CSE is therefore treated as a theoretically relevant correlate rather than a proven causal mechanism.

### 2.2 Indirect role of context-specific learning engagement

Learning engagement refers to behavioral participation, emotional involvement, and cognitive investment (Fredricks et al., 2004; Wu et al., 2025). The questionnaire assessed engagement in the focal SIM-E activities rather than a context-neutral disposition. This contextual specificity is relevant to the research question, but it also creates wording overlap between the SIM-E and LE scales, especially for LE1, LE5, LE8, and LE9. The overlap is identified in the Measurement section and Appendix A and is treated as a substantive limitation on the interpretation of H3.

Context-specific engagement is associated with IC when students sustain attention, interpret feedback, compare alternatives, and revise their work. These processes are relevant to creative production, but the present analysis can show only that the SIM-E composite, the LE composite, and IC covary in the proposed pattern.

The proposed indirect association does not assume that every STEAM activity is engaging. Projects can be superficial, confusing, or overly technical. The focal SIM-E scale therefore captures perceived integration, inquiry, technology use, and creative production rather than course availability alone. Aguilera and Ortiz-Revilla (2021) characterize the creativity evidence as heterogeneous in a systematic review, while Perignat and Katz-Buonincontro (2019) document variation in STEAM definitions and practices.

### 2.3 Parallel indirect associations and an exploratory ordered check

CSE and LE are conceptually related but not interchangeable. Creative self-efficacy concerns belief in one’s creative capability, whereas learning engagement concerns behavioral, emotional, and cognitive investment in ongoing activity. The primary model therefore estimates their specific indirect associations in parallel. This specification aligns the theoretical framework with what the cross-sectional design can support: distinct portions of covariance rather than a developmental chain.

A CSE → LE ordering remains theoretically plausible because confidence may accompany greater willingness to invest effort, tolerate uncertainty, and revise ideas. However, the reverse ordering is also plausible: sustained participation and successful task completion may strengthen creative confidence. The present data cannot adjudicate between these temporal possibilities. Accordingly, the ordered CSE → LE path is retained only as an exploratory sensitivity check and is not used to define the core theoretical model.

The exploratory ordered indirect estimate is evaluated with a 5,000-sample bootstrap confidence interval, but its value is not used to define the core theoretical model. A 95% bootstrap interval excluding zero indicates only that the ordered product differs from zero; it cannot establish that CSE temporally precedes LE when all variables are measured simultaneously by self-report. The primary interpretation therefore remains centered on the two parallel CSE and LE routes.

### 2.4 Hypotheses

The literature supports three directional hypotheses for the observed-score associations. The possible CSE → LE ordering is posed separately as an exploratory research question so that the theoretical framework does not imply a temporal mechanism that the design cannot establish.

*H1*: SIM-E is positively associated with students’ innovative competence.

*H2*: The association between SIM-E and students’ innovative competence includes a positive specific indirect association through CSE.

*H3*: The association between SIM-E and students’ innovative competence includes a positive specific indirect association through context-specific LE.

*RQ1 (exploratory)*: Does the observed covariance pattern provide bootstrap support for an ordered SIM-E → CSE → LE → IC indirect association?

## 3 Materials and methods

A cross-sectional questionnaire design was used. Data were collected from September to December 2025 in Shandong Province, China. The participating institutions had implemented interdisciplinary music, digital-music, or STEAM-related activities before recruitment. The design permitted an initial test with a heterogeneous sample but could not establish temporal or causal order; all structural paths are therefore interpreted as predictive associations.

Convenience sampling was adopted. Five educational institutions participated: two public middle schools, one private middle school, one comprehensive university, and one professional art university. These institutions had offered interdisciplinary music activities, digital music projects, or STEAM-related courses. The sampling strategy was designed to include students from different educational stages and majors, thereby increasing heterogeneity within the available sample. However, the sample should not be treated as nationally representative.

### 3.1 Participants and data collection

Participants were students who had access to music learning contexts in which STEAM-related elements were present. The focal SIM-E scale measured the degree to which students perceived their music-learning experience as interdisciplinary, inquiry-based, technologically supported, and creatively oriented. The binary prior STEAM experience variable, used as a control, referred to whether students had participated in additional STEAM-related courses or activities before or outside the focal music-learning context. This distinction avoids treating exposure to the focal learning environment and prior experience as the same variable.

A total of 520 questionnaires were distributed in paper and online formats, and 487 were returned. Sixty returned questionnaires failed at least one initial quality-screening criterion—missing focal-item data, patterned or invariant responding, or an implausibly short completion time—and were excluded. The archived screening log retained only the combined exclusion count and did not preserve the original item-missingness threshold, missingness-specific respondent or item counts, overlap among screening criteria, or cell-level provenance. Accordingly, those initial-stage quantities cannot be reconstructed without speculation. The analytic sample comprised 427 students, an effective response rate of 82.1%. Digital music tool use and school type were retained for sample description. Because they fell outside the theory-specified control set and some subgroup cells were small, they were excluded from the primary model and entered only in the expanded-control sensitivity analysis reported in Section 4.4.

### 3.2 Measurement

All scales used 5-point Likert-type response options ranging from 1 (strongly disagree) to 5 (strongly agree). To reduce translation bias, the items were translated and back-translated by bilingual researchers familiar with music education and educational psychology. Three scholars reviewed the items for content relevance and linguistic clarity. A pilot test with 30 students was conducted before the formal survey, and several expressions were refined based on feedback.

Because no established music-specific SIM-E instrument was available, nine items were developed from the stated construct definition and reviewed by three experts in music education and educational psychology. Every SIM-E item anchors a general STEAM practice to music learning, a musical problem or product, musical sound or structure, or musical expression. Higher scores therefore indicate stronger perceived implementation of STEAM principles within a music-organized setting, not a uniquely musical mechanism. CSE was assessed with six items adapted from the creative self-efficacy literature (Beghetto, 2006; Tierney and Farmer, 2002). LE was assessed with nine items covering behavioral, emotional, and cognitive participation in the focal context (Fredricks et al., 2004; Schaufeli et al., 2002). Four items—LE1, LE5, LE8, and LE9—explicitly referenced SIM-E activities or projects and therefore posed a plausible shared-wording risk. A reduced five-item LE composite (LE-R: LE2, LE3, LE4, LE6, and LE7) was used in the reviewer-requested sensitivity analysis described in Section 3.5. IC was assessed with seven items addressing idea generation, evaluation, implementation, and transfer (Chen et al., 2024; Runco and Acar, 2012). Appendix A reproduces all analyzed items and marks the four context-overlapping LE items.

The primary control variables were gender, age, years of prior music learning, and prior participation in STEAM-related activities. Gender and prior STEAM experience were coded as binary variables; age and prior music learning were treated as continuous variables. Music-major status and private-school status were binary, and digital-music-tool use was coded ordinally from low to high. The latter three variables were not part of the primary adjustment set; they were added only in an expanded-control sensitivity analysis to determine whether the main indirect estimates depended on the original covariate specification.

### 3.3 Ethical considerations and common method bias control

The study was approved by the Institutional Review Board of Shandong Management University (Approval No. SDMU-20250629; 29 June 2025). Participation was voluntary and responses were anonymous. Adult participants provided informed consent on their own behalf. Minor participants provided assent, and informed consent was obtained from a parent or legal guardian in accordance with the approved protocol. No next-of-kin consent procedure was used.

All focal variables came from the same questionnaire and respondent, creating a potential risk of common method bias (Podsakoff et al., 2003). Same-source measurement is therefore retained as an explicit limitation even though the one-factor model fit substantially worse than the theorized four-factor model.

### 3.4 Data analysis

Analyses proceeded in eight stages. First, the final analysis file was checked for missing values, item distributions, internal consistency, descriptive statistics, observed-score correlations, and variance-inflation factors (VIFs). Second, a maximum-likelihood confirmatory factor analysis (CFA) was fitted to the 31 focal items, specifying SIM-E, CSE, LE, and IC as correlated latent variables with no cross-loadings or correlated residuals. The four-factor model was compared with two theoretically plausible three-factor models that collapsed SIM-E with LE or CSE with LE, and with a one-factor model. Model evaluation used *χ*^2^, df, *χ*^2^/df, CFI, TLI, RMSEA with 90% confidence interval, and SRMR, interpreted jointly (Hu and Bentler, 1999). Third, standardized loadings, composite reliability (CR), average variance extracted (AVE), latent-factor correlations, and the heterotrait-monotrait ratio (HTMT) were used to evaluate convergent and discriminant validity (Fornell and Larcker, 1981; Henseler et al., 2015). Fourth, multigroup CFA across middle-school and university participants evaluated configural, metric, and scalar invariance. Changes in CFI and RMSEA were considered together with *χ*^2^-difference tests, following the practical guidance of Chen (2007). Fifth, the primary observed-score model estimated CSE and LE as parallel indirect variables while controlling for gender, age, prior music experience, and prior STEAM experience; variables were standardized so that reported path coefficients are *β* coefficients. Sixth, specific indirect associations were evaluated with 5,000 nonparametric percentile-bootstrap resamples (Preacher and Hayes, 2008; Hayes, 2018). Seventh, LE1, LE5, LE8, and LE9 were removed to form LE-R = mean (LE2, LE3, LE4, LE6, LE7), after which the CFA and indirect-association analyses were repeated as a targeted wording-overlap sensitivity test. Eighth, robustness was examined by adding music-major status, digital-music-tool use, and school type to the adjustment set and by estimating the parallel indirect associations separately in the two educational-stage groups with 5,000 bootstrap resamples.

Missing-data reporting was separated by stage (Table 1). At the initial screening stage, the precise item-missingness threshold and the numbers of respondents and item responses affected specifically by missingness are not recoverable from the archived log; the manuscript therefore reports these quantities as unavailable rather than assigning unverifiable values. For the reported reanalysis, the operational rule was complete-case analysis across the 31 focal items: any missing focal-item response would exclude the case (allowable threshold = 0/31). All 427 retained cases had recorded values on all 31 focal items, yielding 13,237/13,237 recorded focal-item values; therefore, 0/427 respondents and 0/13,237 focal-item responses required exclusion or new item-level imputation at reanalysis. Composite scores required all constituent items: SIM-E 9/9, CSE 6/6, LE 9/9, IC 7/7, and LE-R 5/5. No person-mean substitution was performed during the reported reanalysis. Because the archived file does not preserve pre-archive cell-level provenance, this audit cannot establish whether any values had been replaced before archiving.

**Table 1:** Missing-data audit and composite-score requirements.

Stage	Rule or threshold	Affected respondents/item responses	Composite-score requirement and imputation status
Initial screening of 487 returns	Failed ≥1 quality criterion; original item-missingness threshold not recoverable	60 failed ≥1 criterion; missingness-specific respondent and item counts not recoverable	Original pre-archive replacement provenance and criterion overlap not preserved
Archived reanalysis file (*n* = 427)	Complete case across 31 focal items; allowable missingness = 0/31	0/427 respondents; 0/13,237 focal-item responses	SIM-E 9/9; CSE 6/6; LE 9/9; IC 7/7; LE-R 5/5; no new item-level imputation

The table separates verifiable reanalysis-stage quantities from initial-stage information that cannot be reconstructed from the archived screening materials. “No new item-level imputation” refers only to the reported reanalysis and does not establish the provenance of values before the archived file was created.

The exploratory CSE → LE ordered product was estimated separately from the primary parallel model. The same 5,000 bootstrap resamples were used for the full-LE and reduced-LE-R analyses, allowing a paired bootstrap confidence interval for the change in the LE-specific indirect estimate. For the stage-specific analysis, participants were resampled within educational stage and bootstrap intervals were calculated for each indirect estimate and for the middle-school-minus-university difference. A 95% bootstrap interval excluding zero was used as the statistical criterion for an indirect association or between-group difference. An ordered product whose 95% bootstrap interval excluded zero was recorded as nonzero; because all focal variables were measured at the same occasion, a nonzero product was interpreted only as a compatible covariance pattern and not as evidence of temporal precedence or a causal sequence.

### 3.5 Measurement validation and robustness criteria

The central robustness criterion concerned possible construct contamination of LE by context-specific wording. The full nine-item LE composite was retained as the primary measure. The reduced-item analysis was a reviewer-requested sensitivity analysis for which the evaluation criteria were specified before the reanalysis was conducted. LE1, LE5, LE8, and LE9 were removed because the engaged behavior they described duplicated instructional practices measured by the SIM-E scale: participation in SIM-E activities, feedback during STEAM music projects, collaboration in SIM-E projects, and revising SIM-E work. LE3 remained because careful completion of assigned tasks is an engagement indicator even though the item names the focal activities. H3 was treated as robust only if (a) the SIM-E → LE-R and LE-R → IC paths remained directionally consistent, (b) the SIM-E → LE-R → IC 95% bootstrap interval excluded zero, and (c) the reduced-item indirect estimate did not materially attenuate relative to the full-LE estimate. A paired bootstrap interval for the difference between the two indirect estimates provided an additional sensitivity check.

Construct validity was evaluated before interpreting structural paths. The four-factor CFA, standardized loadings, CR, AVE, latent correlations, and HTMT were considered jointly. The one-factor CFA served as a same-source diagnostic, and multigroup CFA evaluated whether the measurement structure was sufficiently comparable across educational stages. At the observed-score level, VIFs were inspected as a supplementary collinearity diagnostic. Finally, the reduced-item, expanded-control, and stage-specific analyses were treated as robustness checks rather than as new confirmatory hypotheses. These analyses strengthen measurement and sensitivity evidence but do not convert the cross-sectional self-report design into a causal mediation design (Table 2).

**Table 2:** Demographic characteristics of participants (*N* = 427).

Variable	Category	*n*	%
Gender	Male	212	49.6
	Female	215	50.4
Age	14–17	187	43.8
	18–21	205	48.0
	22+	35	8.2
Educational stage	Middle school	189	44.3
	University	238	55.7
Major	Music major	156	36.5
	Non-music major	271	63.5
Prior STEAM-related activity	Yes	198	46.4
	No	229	53.6
Digital music tool use	High	124	29.0
	Medium	207	48.5
	Low	96	22.5
School type	Public	352	82.4
	Private	75	17.6

Digital music tool use and school type were not included in the primary model; together with music-major status, they were used only in the expanded-control sensitivity analysis.

## 4 Results

The archived analysis file contained 427 cases with recorded values for all 31 focal items (13,237/13,237 item values; Table 1). Under the complete-case reanalysis rule described in Section 3.4, no participant required exclusion and no new item-level imputation was performed. Because the pre-archive screening log did not preserve cell-level provenance, these statements describe the archived reanalysis file and do not retrospectively establish whether any values had been replaced during the earlier data-cleaning stage. Descriptive and composite diagnostics were followed by latent measurement-model checks, the same-source diagnostic, the shared-wording sensitivity analysis, the primary parallel indirect model, and the exploratory ordered product.

### 4.1 Descriptive statistics and composite-score diagnostics

Table 3 reports means, standard deviations, skewness, and kurtosis. The focal composite means ranged from 3.31 to 3.42 on the 5-point response scale: SIM-E (*M* = 3.36, SD = 0.82), CSE (*M* = 3.42, SD = 0.78), LE (*M* = 3.31, SD = 0.81), and IC (*M* = 3.38, SD = 0.82). The reduced five-item LE-R composite used in sensitivity analysis had *M* = 3.31 and SD = 0.88. Prior STEAM experience was binary coded (*M* = 0.46, SD = 0.50). Table 4 reports the observed Pearson correlations among the controls and focal composite scores.

**Table 3:** Means, standard deviations, skewness, and kurtosis (*N* = 427).

Variable	*M*	SD	Skewness	Kurtosis
1. Gender	0.50	0.50	−0.01	−2.01
2. Age	17.73	2.32	0.36	−0.09
3. Prior music experience	4.15	3.62	0.92	0.19
4. Prior STEAM experience	0.46	0.50	0.15	−1.99
5. SIM-E	3.36	0.82	−0.15	−0.22
6. CSE	3.42	0.78	0.10	−0.20
7. LE	3.31	0.81	−0.21	0.12
8. IC	3.38	0.82	0.23	0.02

Gender and prior STEAM experience were binary-coded variables; all focal constructs were measured on 5-point scales.

**Table 4:** Pearson correlation matrix for observed composite scores (*N* = 427).

Variable	1	2	3	4	5	6	7	8
1. Gender	1							
2. Age	0.04	1						
3. Prior music experience	0.02	0.30^***^	1					
4. Prior STEAM experience	−0.03	0.14^**^	0.13^**^	1				
5. SIM-E	0.03	−0.05	0.12^*^	0.24^***^	1			
6. CSE	0.02	−0.02	0.12^*^	0.21^***^	0.46^***^	1		
7. LE	0.03	−0.01	0.10^*^	0.18^***^	0.27^***^	0.38^***^	1	
8. IC	0.05	0.05	0.21^***^	0.20^***^	0.37^***^	0.39^***^	0.43^***^	1

^*^p < 0.05, ^**^p < 0.01, ^***^p < 0.001.

The focal construct correlations were positive and ranged from 0.273 to 0.464: SIM-E correlated with CSE (*r* = 0.464), LE (*r* = 0.273), and IC (*r* = 0.374); CSE correlated with LE (*r* = 0.382) and IC (*r* = 0.385); and LE correlated with IC (*r* = 0.434), all *p* < 0.001. These are descriptive observed-score associations and are not interpreted as latent-factor correlations or as a Fornell–Larcker matrix.

Internal consistency was acceptable for SIM-E (*α* = 0.862), CSE (*α* = 0.881), LE (*α* = 0.847), and IC (*α* = 0.829); the reduced five-item LE-R composite had *α* = 0.752 (Table 5).

**Table 5:** Internal consistency of the composite scales.

Construct	Items	Cronbach’s *α*
SIM-E	9	0.862
CSE	6	0.881
LE	9	0.847
IC	7	0.829
LE-R	5	0.752

*α* = Cronbach’s alpha. LE-R is the five-item reduced engagement composite used only for sensitivity analysis.

The primary path estimates remain relations among observed composite scores. The CFA supports empirical separation of the constructs, but the regression and bootstrapping results are not interpreted as latent structural effects or as causal mediation. VIFs for SIM-E, CSE, LE, and the four primary controls ranged from 1.006 to 1.414, indicating that the reported regression coefficients were not generated under severe observed-score collinearity.

### 4.2 Confirmatory measurement model and construct validity

The four-factor CFA showed good fit, *χ*^2^(428) = 489.87, *χ*^2^/df = 1.14, CFI = 0.986, TLI = 0.985, RMSEA = 0.018 (90% CI [0.008, 0.026]), and SRMR = 0.038 (Table 6). Both collapsed three-factor models fit substantially worse: combining SIM-E with LE yielded CFI = 0.801, TLI = 0.785, RMSEA = 0.070, and SRMR = 0.089; combining CSE with LE yielded CFI = 0.832, TLI = 0.819, RMSEA = 0.064, and SRMR = 0.076. These comparisons support factorial separation of the four constructs. Standardized loadings were 0.592–0.690 for SIM-E, 0.735–0.750 for CSE, 0.564–0.653 for LE, and 0.618–0.661 for IC, and CR ranged from 0.829 to 0.881. Convergent-validity evidence was mixed: AVE was 0.553 for CSE but 0.411 for SIM-E, 0.382 for LE, and 0.409 for IC, so the strict AVE ≥ 0.50 criterion was met only for CSE. Discriminant-validity evidence was supported separately: latent correlations ranged from 0.316 to 0.534, each construct’s square root of AVE exceeded its largest latent correlation, and pairwise HTMT values ranged from 0.319 to 0.533, below commonly used 0.85/0.90 reference values. Table 7 summarizes these distinct forms of evidence.

**Table 6:** Confirmatory factor-analysis model comparison.

Model	*χ* ^2^	df	*χ*^2^/df	CFI	TLI	RMSEA (90% CI)	SRMR
Four-factor: SIM-E, CSE, LE, IC	489.87	428	1.14	0.986	0.985	0.018 [0.008, 0.026]	0.038
Three-factor: SIM-E + LE combined	1336.22	431	3.10	0.801	0.785	0.070 [0.066, 0.075]	0.089
Three-factor: CSE + LE combined	1192.29	431	2.77	0.832	0.819	0.064 [0.060, 0.069]	0.076
One-factor: all focal items	2249.27	434	5.18	0.601	0.572	0.099 [0.095, 0.103]	0.109

Maximum-likelihood CFA of the 31 focal items. RMSEA values are accompanied by 90% confidence intervals. The one-factor model is used as a same-source/common-method diagnostic; its poor fit does not by itself prove the absence of common-method variance.

**Table 7:** Construct reliability and validity from the four-factor CFA.

Construct	Std. loading range	CR	AVE	Maximum HTMT with another construct	Interpretation
SIM-E	0.592–0.690	0.862	0.411	0.533	CR adequate; AVE criterion not met; discriminant validity supported
CSE	0.735–0.750	0.881	0.553	0.533	AVE criterion met; discriminant validity supported
LE	0.564–0.653	0.847	0.382	0.517	CR adequate; AVE criterion not met; discriminant validity supported
IC	0.618–0.661	0.829	0.409	0.517	CR adequate; AVE criterion not met; discriminant validity supported

CR, composite reliability; AVE, average variance extracted; HTMT, heterotrait-monotrait ratio. Factorial separation was supported by the four-factor versus collapsed-factor comparisons in Table 6. Convergent validity, evaluated with AVE, met the strict AVE ≥ 0.50 criterion only for CSE. Discriminant validity, evaluated separately, was supported by HTMT and Fornell-Larcker evidence (Fornell and Larcker, 1981; Henseler et al., 2015).

### 4.3 Same-source/common-method diagnostic

The one-factor CFA fit poorly, *χ*^2^(434) = 2249.27, *χ*^2^/df = 5.18, CFI = 0.601, TLI = 0.572, RMSEA = 0.099 (90% CI [0.095, 0.103]), and SRMR = 0.109. Its fit was markedly inferior to the four-factor model. This result is inconsistent with a single common factor accounting for the item covariance, but it does not rule out more limited forms of same-source/common-method variance; that risk remains a design limitation.

### 4.4 Sensitivity analysis for context-overlapping LE items

Deleting LE1, LE5, LE8, and LE9 produced LE-R (five items; *α* = 0.752). A four-factor CFA using LE-R also fit well, *χ*^2^(318) = 365.26, CFI = 0.988, TLI = 0.987, RMSEA = 0.019 (90% CI [0.006, 0.027]), and SRMR = 0.037. In the primary parallel observed-score model, the SIM-E → LE path was *β* = 0.233 (SE = 0.048, *p* < 0.001) with the full LE measure and *β* = 0.226 (SE = 0.049, *p* < 0.001) with LE-R. The LE/LE-R → IC coefficients were *β* = 0.300 and *β* = 0.298, respectively (both *p* < 0.001). The LE-specific indirect estimate was 0.070 (bootstrap SE = 0.017, 95% CI [0.038, 0.106]) with the full measure and 0.067 (bootstrap SE = 0.017, 95% CI [0.035, 0.103]) with LE-R. The point estimate decreased by only 0.003 (4.1%), and the paired bootstrap interval for the full-minus-reduced difference included zero, 95% CI [−0.010, 0.016]. H3 therefore remained supported after removing the four context-overlapping items. The exploratory ordered product was similarly stable, 0.040 (95% CI [0.024, 0.060]) with full LE and 0.043 (95% CI [0.026, 0.062]) with LE-R. A second sensitivity analysis added music-major status, digital-music-tool use, and school type to the four primary controls. With the full LE measure, the CSE-specific indirect estimate was 0.072 (95% CI [0.032, 0.117]) and the LE-specific estimate was 0.069 (95% CI [0.038, 0.104]); with LE-R, the corresponding estimates were 0.069 (95% CI [0.031, 0.114]) and 0.066 (95% CI [0.034, 0.102]). Thus, the two focal indirect associations were also stable to the expanded covariate specification (Table 8).

**Table 8:** Sensitivity analysis for context-overlapping learning-engagement items.

Analysis	Full 9-item LE result	Reduced 5-item LE-R result	Robustness decision
SIM-E → LE/LE-R	*β* = 0.233, SE = 0.048, *p* < 0.001	*β* = 0.226, SE = 0.049, *p* < 0.001	Stable
LE/LE-R → IC	*β* = 0.300, SE = 0.045, *p* < 0.001	*β* = 0.298, SE = 0.045, *p* < 0.001	Stable
SIM-E → LE/LE-R → IC	0.070, SE = 0.017, 95% CI [0.038, 0.106]	0.067, SE = 0.017, 95% CI [0.035, 0.103]	Robust; Δ = 0.003, 95% CI [−0.010, 0.016]
SIM-E → CSE → LE/LE-R → IC (exploratory)	0.040, SE = 0.009, 95% CI [0.024, 0.060]	0.043, SE = 0.009, 95% CI [0.026, 0.062]	Nonzero and stable; not temporal evidence
Expanded controls: SIM-E → CSE → IC	0.072, 95% CI [0.032, 0.117]	0.069, 95% CI [0.031, 0.114]	Stable under expanded covariate set
Expanded controls: SIM-E → LE/LE-R → IC	0.069, 95% CI [0.038, 0.104]	0.066, 95% CI [0.034, 0.102]	Stable under expanded covariate set

LE-R = mean of LE2, LE3, LE4, LE6, and LE7 after removing LE1, LE5, LE8, and LE9. Indirect-effect confidence intervals are percentile intervals from 5,000 paired bootstrap resamples. The paired full-minus-reduced difference for the LE-specific indirect effect was 0.003, 95% CI [−0.010, 0.016]. Expanded controls add music-major status, digital-music-tool use, and school type to gender, age, prior music experience, and prior STEAM experience.

### 4.5 Educational-stage measurement invariance and multigroup robustness

Multigroup CFA indicated that the four-factor measurement structure was comparable across middle-school (*n* = 189) and university (*n* = 238) participants (Table 9). The configural model fit well, *χ*^2^(856) = 971.07, CFI = 0.975, TLI = 0.973, RMSEA = 0.018. Constraining factor loadings to equality did not worsen fit, *χ*^2^(883) = 988.03, CFI = 0.977, RMSEA = 0.017; Δ*χ*^2^(27) = 16.96, *p* = 0.932, ΔCFI = +0.002, and ΔRMSEA = −0.001. Adding equality constraints on item intercepts likewise produced little change, *χ*^2^(910) = 1014.39, CFI = 0.977, RMSEA = 0.016; Δ*χ*^2^(27) = 26.36, *p* = 0.499, ΔCFI < 0.001, and ΔRMSEA < 0.001 in absolute value. These results support configural, metric, and scalar invariance for the four-factor measurement model across the two educational stages.

**Table 9:** Educational-stage measurement invariance of the four-factor CFA.

Model	*χ* ^2^	df	CFI	TLI	RMSEA	ΔCFI	ΔRMSEA
Configural	971.07	856	0.975	0.973	0.018	–	–
Metric	988.03	883	0.977	0.976	0.017	+0.002	−0.001
Scalar	1014.39	910	0.977	0.977	0.016	+0.000	−0.000

Groups: middle school *n* = 189; university *n* = 238. Metric versus configural: Δ*χ*^2^(27) = 16.96, *p* = 0.932. Scalar versus metric: Δ*χ*^2^(27) = 26.36, *p* = 0.499. ΔCFI and ΔRMSEA are changes from the immediately less constrained model. Fit-index changes were interpreted jointly with *χ*^2^-difference tests following Chen (2007).

Stage-specific observed-score bootstrap analyses were then used as an exploratory robustness check rather than as a formal moderation hypothesis (Table 10). In the middle-school group, the CSE-specific indirect estimate was 0.088 (95% CI [0.031, 0.156]) and the LE-specific estimate was 0.080 (95% CI [0.028, 0.142]). In the university group, the corresponding estimates were 0.045 (95% CI [−0.012, 0.106]) and 0.059 (95% CI [0.021, 0.105]). Although the university CSE-specific interval included zero, the bootstrap confidence intervals for the between-stage differences included zero for both the CSE route, Δ = 0.043, 95% CI [−0.039, 0.132], and the LE route, Δ = 0.021, 95% CI [−0.048, 0.093]. The direct-association difference also included zero, Δ = −0.084, 95% CI [−0.270, 0.098]. Accordingly, the data do not provide clear evidence that the focal parallel associations differ by educational stage, although the group-specific CSE estimate is less precise among university students.

**Table 10:** Educational-stage bootstrap robustness of the primary parallel associations.

Association	Middle school (*n* = 189)	University (*n* = 238)	Difference: middle—university
SIM-E → CSE → IC	0.088 [0.031, 0.156]	0.045 [−0.012, 0.106]	0.043 [−0.039, 0.132]
SIM-E → LE → IC	0.080 [0.028, 0.142]	0.059 [0.021, 0.105]	0.021 [−0.048, 0.093]
Direct SIM-E → IC	0.157 [0.017, 0.290]	0.241 [0.117, 0.361]	−0.084 [−0.270, 0.098]

Entries are standardized point estimates with 95% percentile bootstrap confidence intervals from 5,000 within-stage resamples. Difference intervals are based on the bootstrap distribution of the middle-school-minus-university estimate. The stage-specific analysis is exploratory and does not establish developmental moderation.

### 4.6 Hypothesis 1: direct association between SIM-E and innovative competence

After adjustment for gender, age, prior music experience, and prior STEAM experience, the total SIM-E–IC association was positive (*β* = 0.331, model-based SE = 0.046, *p* < 0.001; 95% bootstrap CI [0.243, 0.413]). After CSE and LE were entered simultaneously, the residual direct association remained positive (*β* = 0.194, model-based SE = 0.047, *p* < 0.001; 95% bootstrap CI [0.101, 0.280]), representing 58.6% of the total association. Prior music experience was also positively associated with IC (*β* = 0.120, SE = 0.044, *p* = 0.006); the other IC controls were not significant. H1 was supported at the observed-score level.

Table 11 reports the primary parallel-model coefficients, and Table 12 reports the 5,000-resample bootstrap estimates for the direct, total, and specific indirect associations. All coefficients describe conditional associations among observed scores.

**Table 11:** Standardized composite-score path coefficients.

Predictor	Outcome	*β*	SE	*p*
Primary parallel model				
SIM-E	CSE	0.430	0.045	< 0.001
SIM-E	LE	0.233	0.048	< 0.001
SIM-E	IC	0.194	0.047	< 0.001
CSE	IC	0.155	0.049	0.002
LE	IC	0.300	0.045	< 0.001
Gender	CSE	0.010	0.043	0.820
Gender	LE	0.023	0.047	0.617
Gender	IC	0.030	0.041	0.462
Age	CSE	−0.040	0.045	0.381
Age	LE	−0.042	0.049	0.392
Age	IC	0.020	0.044	0.640
Prior music experience	CSE	0.070	0.045	0.124
Prior music experience	LE	0.072	0.049	0.147
Prior music experience	IC	0.120	0.044	0.006
Prior STEAM experience	CSE	0.100	0.045	0.025
Prior STEAM experience	LE	0.121	0.048	0.013
Prior STEAM experience	IC	0.050	0.043	0.251
Exploratory ordered-model addition (estimated separately)				
CSE	LE	0.310	0.051	< 0.001

*β* = standardized coefficient. SEs are model-based regression standard errors; Table 12 reports bootstrap standard errors and percentile confidence intervals. Panel A contains only the primary parallel-model equations: the CSE and LE equations each include SIM-E and the four controls, and the IC equation includes SIM-E, CSE, LE, and the four controls simultaneously. Panel B reports the additional CSE → LE coefficient from a separately estimated exploratory ordered model. It is not part of the primary parallel specification.

**Table 12:** Bootstrap indirect associations.

Path	Estimate	Bootstrap SE	95% CI	Proportion (%)
Total indirect association (parallel)	0.137	0.026	[0.088, 0.190]	41.4
SIM-E → CSE → IC	0.067	0.021	[0.028, 0.111]	20.2
SIM-E → LE → IC	0.070	0.017	[0.038, 0.106]	21.2
SIM-E → CSE → LE → IC (exploratory)	0.040	0.009	[0.024, 0.060]	—
Direct association (parallel model)	0.194	0.046	[0.101, 0.280]	58.6
Total association	0.331	0.044	[0.243, 0.413]	100.0

Bootstrap SEs and percentile 95% CIs are based on 5,000 resamples. H2 and H3 are the two primary parallel indirect associations. The exploratory ordered SIM-E → CSE → LE → IC product was nonzero, 95% bootstrap interval [0.024, 0.060]. It is reported as an exploratory covariance pattern and is not interpreted as evidence of temporal sequencing.

### 4.7 Hypothesis 2: specific indirect association through creative self-efficacy

SIM-E was positively associated with CSE (*β* = 0.430, SE = 0.045, *p* < 0.001), and CSE was positively associated with IC after SIM-E, LE, and the controls were included (*β* = 0.155, SE = 0.049, *p* = 0.002). The CSE-specific indirect estimate was 0.067 (bootstrap SE = 0.021, 95% CI [0.028, 0.111]), equivalent to 20.2% of the total SIM-E–IC association. Because the interval excluded zero, H2 was supported.

### 4.8 Hypothesis 3: specific indirect association through context-specific learning engagement

In the primary parallel model, SIM-E was positively associated with LE (*β* = 0.233, SE = 0.048, *p* < 0.001), and LE was positively associated with IC after SIM-E, CSE, and the controls were included (*β* = 0.300, SE = 0.045, *p* < 0.001). The LE-specific indirect estimate was 0.070 (bootstrap SE = 0.017, 95% CI [0.038, 0.106]), equivalent to 21.2% of the total association. As reported in Section 4.4, the reduced-item LE-R estimate remained 0.067 (95% CI [0.035, 0.103]), and the paired change was small and statistically indistinguishable from zero. H3 was therefore supported and was robust to removal of the four most context-overlapping LE items.

### 4.9 Exploratory ordered indirect association (RQ1)

The ordered model was treated as exploratory rather than as a fourth directional hypothesis. In that parameterization, SIM-E was associated with CSE (*β* = 0.430, SE = 0.045, *p* < 0.001), CSE was positively associated with LE after SIM-E and the controls were included (*β* = 0.310, SE = 0.051, *p* < 0.001), and LE was associated with IC (*β* = 0.300, SE = 0.045, *p* < 0.001). The ordered SIM-E → CSE → LE → IC product was 0.040 (bootstrap SE = 0.009, 95% CI [0.024, 0.060]), so its bootstrap interval excluded zero, and the product remained 0.043 (95% CI [0.026, 0.062]) with LE-R. RQ1 therefore yielded a nonzero ordered covariance product. In the reduced-item parameterization, the residual direct SIM-E → LE-R coefficient after CSE was included was *β* = 0.083 (SE = 0.051, *p* = 0.108); this coefficient concerns only the residual SIM-E → LE-R path and does not itself alter the ordered product. Because the variables were measured simultaneously, the analysis cannot establish that CSE temporally precedes LE. The ordered result is therefore reported as exploratory compatibility with that ordering, not as evidence for a sequential psychological mechanism.

### 4.10 Summary of hypothesis tests and robustness decisions

H1, H2, and H3 were supported at the observed-score level in the full sample. The four-factor CFA and low HTMT values supported empirical separation of SIM-E, CSE, LE, and IC, although AVE below 0.50 for SIM-E, LE, and IC requires caution about convergent validity. Configural, metric, and scalar invariance were supported across middle-school and university participants. The LE-specific indirect estimate remained statistically significant after removing LE1, LE5, LE8, and LE9, and both primary indirect estimates remained statistically significant after adding music-major status, digital-music-tool use, and school type to the covariate set; in each case, the 95% bootstrap confidence interval excluded zero. Stage-specific bootstrap difference intervals included zero, providing no clear evidence of educational-stage moderation, while also showing greater uncertainty for the university CSE-specific estimate. For RQ1, the exploratory ordered product was nonzero (0.040, 95% CI [0.024, 0.060]). It is treated as a covariance pattern compatible with the proposed ordering and not as evidence of temporal sequencing, because the data are cross-sectional. The theoretical conclusion therefore remains a parallel-association account rather than a confirmed sequential mediation account.

## 5 Discussion

The four-factor CFA outperformed the collapsed-factor and one-factor alternatives, supporting factorial separation among SIM-E, CSE, LE, and IC. Discriminant validity was supported by the latent-correlation comparisons and HTMT values. Convergent-validity evidence was mixed rather than uniformly strong because AVE remained below 0.50 for SIM-E, LE, and IC. Within this measurement context, SIM-E showed a positive adjusted total association with IC, CSE and LE each showed distinct positive specific indirect associations, and the residual direct association remained positive after both variables were included.

The additional robustness checks sharpen this interpretation. First, scalar measurement invariance indicates that the four constructs were represented comparably across the middle-school and university groups at the level of factor loadings and item intercepts. Second, neither primary indirect association disappeared when music-major status, digital-music-tool use, and school type were added to the adjustment set. Third, bootstrap intervals for middle-school-minus-university differences included zero for both indirect routes. This does not establish equality of mechanisms across developmental stages, especially because the university CSE-specific interval itself crossed zero, but it reduces the likelihood that the full-sample pattern is an artifact of only one educational-stage subgroup.

The direct SIM-E-IC coefficient was modest, consistent with evidence that STEAM outcomes depend on implementation rather than on a uniform intervention effect. Systematic reviews report heterogeneous creativity findings and substantial variation in STEAM definitions and practices (Aguilera and Ortiz-Revilla, 2021; Perignat and Katz-Buonincontro, 2019). The present result should therefore be read as an association with students’ perceptions of one instructional context, not as evidence that music-centered STEAM is superior to other STEAM approaches.

The CSE-specific and LE-specific indirect estimates were similar in magnitude (0.067 and 0.070, respectively). The CSE result is consistent with the role of mastery experiences, peer models, feedback, and visible progress in creative confidence. Prior research links creative self-efficacy with creative performance and school students’ creative beliefs (Tierney and Farmer, 2002; Beghetto, 2006; Puente-Díaz, 2016). The present data extend that pattern to a music-organized STEAM context without establishing that SIM-E causes changes in CSE.

The LE-specific estimate points to a second, theoretically distinct route involving participation and cognitive-emotional investment. Recent qualitative evidence from Chinese university music classes further suggests that teacher scaffolding may support learner autonomy, collaboration, motivation, disciplinary learning, and creativity while requiring attention to learner diversity and the timing of support (Li and Wang, 2026). These findings provide a practical basis for emphasizing formative feedback, adaptive task sequencing, and appropriately timed instructional support.

The exploratory ordered SIM-E → CSE → LE → IC product was nonzero in the reanalysis (0.040, 95% CI [0.024, 0.060]), but this does not justify a sequential psychological story. CSE and LE were measured at the same occasion, and reverse ordering is theoretically plausible. The theoretical framework therefore continues to prioritize the two parallel CSE and LE routes. Longitudinal data with temporally separated measurements, cross-lagged or longitudinal mediation models, or experimental manipulation would be needed to distinguish reciprocal, parallel, and ordered processes.

### 5.1 Contribution to general STEAM and educational psychology

The contribution to general STEAM and educational psychology is application-focused. The study does not introduce a new theory of environment-belief-engagement-outcome relations. Instead, it shows that CSE and LE account for distinguishable and similarly sized portions of the SIM-E–IC association and that the LE-specific estimate survives a targeted wording-overlap sensitivity analysis. Although the exploratory ordered product was nonzero, the theoretical contribution remains the parallel account because the design cannot identify temporal precedence. This framing aligns the theory with the measurement-model evidence and the limits of cross-sectional inference.

This pattern is consistent with the study’s theoretical sources: social cognitive theory informs the efficacy-belief route, and engagement theory informs the engaged-state route. Because all variables were measured simultaneously by self-report, neither route can be assigned temporal priority. The study therefore applies established perspectives to a STEAM context rather than claiming a new general mechanism.

### 5.2 Contextual contribution to music-centered STEAM

The contribution to music education is contextual rather than music-specific. The SIM-E construct anchors interdisciplinary inquiry, design, technology use, collaboration, and revision to musical questions, products, sounds, structures, expression, and evaluative criteria. The findings show that the two parallel observed-score associations are present in this setting. They do not show that the associations are caused by music, unique to music, or stronger than those in non-music STEAM contexts.

Future comparative studies should use matched music-centered and non-music STEAM conditions, common outcome measures, and direct indicators of potentially music-specific processes such as improvisation, embodied coordination, esthetic judgment, and auditory feedback. Such designs are needed to distinguish general STEAM processes from effects attributable to the musical context.

### 5.3 Practical implications

The observed associations suggest curriculum priorities rather than tested treatment effects. Music-centered STEAM should begin with authentic musical problems that require inquiry, experimentation, and creative production. Examples include instrument construction for exploring resonance, digital audio modeling of waveform changes, mathematical patterning in rhythm, and collaborative performance design.

Teachers can support creative self-efficacy by sequencing tasks from manageable challenges to open-ended projects, making creative strategies visible, providing formative feedback, and encouraging revision without fear of failure. Peer demonstration and collaborative critique can help students understand creativity as a process. These recommendations align with recent music-education evidence. Li and Wang's (2026) qualitative study of 22 Chinese university music teachers found that appropriately timed scaffolding was perceived to support learner autonomy, collaboration, motivation, disciplinary learning, creativity, and classroom supportiveness, while learner diversity and mistimed support created practical challenges. In a STEAM-oriented art-song context, Chen and Dong (2024) reported gains in teamwork/interpersonal skills and learning interest; because their design, measures, and setting differ from the present study, the finding is used as contextual support rather than as a direct replication.

Engagement can be supported through autonomy, relevance, and collaboration. Students need meaningful choices about materials, roles, tools, or expressive directions within a clear project structure. Digital music tools are most useful when they extend exploration rather than replace musical thinking.

School administrators can support SIM-E through interdisciplinary teaching teams and professional development focused on project design, assessment rubrics, and feedback practices. Evaluation should include creative process, collaboration, and revision rather than visible products alone.

### 5.4 Limitations and future research

The cross-sectional design prevents conclusions about temporal precedence, causal effects, or mediation as a developmental process. Longitudinal studies should separate SIM-E experiences, CSE, LE, and IC across measurement occasions, while intervention studies should compare change with an appropriate control condition.

All focal variables were reported by the same respondents, creating a risk of common-method variance. The poor one-factor CFA and the much better fit of the four-factor model make a single undifferentiated common factor implausible, but such model comparisons cannot establish that common-method bias is absent. Future studies should combine self-reports with teacher ratings, behavioral or performance indicators, or temporally separated measurement.

Convenience sampling from five institutions in one region limits generalizability. The middle-school and university groups also differ in age, educational context, curriculum structure, and likely opportunity to specialize in music. Multigroup CFA supported configural, metric, and scalar invariance, and exploratory bootstrap difference intervals did not show clear stage differences in the two indirect associations. These findings improve comparability but do not establish developmental invariance of the structural process. The university CSE-specific estimate was also less precise and its 95% interval included zero. Future work should recruit independent age-stratified samples across regions and institutions, test structural invariance prospectively, and examine whether school level, music specialization, or instructional format moderates the associations.

The archived reanalysis file was complete, and no person-mean substitution was performed during the reported reanalysis. However, the initial screening log grouped missing-data failures with patterned responding and implausibly short completion times and did not preserve the original missingness threshold, missingness-specific counts, overlap among criteria, or cell-level provenance for archived item values. Consequently, the number of initial exclusions attributable specifically to missingness and the number of respondents or responses, if any, affected by pre-archive person-mean replacement cannot be verified. If person-mean replacement occurred before archiving, it could attenuate variance and underestimate uncertainty. This unresolved provenance is a reproducibility limitation of the initial screening stage, even though the reanalysis-stage rule, denominator, affected count, and item requirements are explicit in Section 3.4 and Table 1. Future studies should retain item-level missingness logs, reason-specific exclusion counts, dated data-cleaning syntax, and an untouched raw-data file; full-information or multiple-imputation methods should be considered when missingness is nontrivial.

## 6 Conclusion

The four-factor CFA supported factorial separation of SIM-E, CSE, LE, and IC, and discriminant validity was supported by factor comparisons and HTMT evidence. However, convergent-validity evidence remained mixed because AVE was below 0.50 for SIM-E, LE, and IC.

The study applies established efficacy and engagement perspectives to a music-organized STEAM context while keeping its claims bounded. Measurement-model comparisons, CR/AVE and HTMT checks, the one-factor diagnostic, educational-stage measurement invariance, the reduced-item analysis, expanded-control sensitivity, and stage-specific bootstrap checks provide converging evidence that the two primary indirect associations are not artifacts of a single simple measurement or specification choice. The exploratory ordered product was nonzero (0.040, 95% CI [0.024, 0.060]). At the same time, the cross-sectional same-source design cannot demonstrate a music-specific effect, causal mediation, developmental invariance, or temporal ordering. The ordered result is therefore retained as a covariance-compatible secondary finding rather than as a confirmed sequential mechanism.

## Data Availability

The raw data supporting the conclusions of this article will be made available by the authors, without undue reservation.

## Appendix A. Measurement items used in the survey

All items were administered in Chinese with 5-point response options from 1 (strongly disagree) to 5 (strongly agree). The English translations below reproduce the analyzed item content. The SIM-E items anchor interdisciplinary inquiry and production to music learning, musical problems or products, sounds, rhythms, structures, expression, or musical evaluation. They operationalize general STEAM practices in a music-organized context rather than uniquely musical mechanisms. The dagger marks LE1, LE5, LE8, and LE9, whose wording explicitly refers to SIM-E activities or projects (Table A1).

**Table A1:** Measurement items.

Construct	Code	Item wording
SIM-E	SIM1	My music learning activities integrate knowledge from music and at least one STEAM-related discipline.
SIM-E	SIM2	Music projects require me to connect musical ideas with scientific, technological, engineering, or mathematical concepts.
SIM-E	SIM3	Teachers encourage us to solve music-related problems through interdisciplinary inquiry.
SIM-E	SIM4	Digital music tools or technological resources are used meaningfully in music learning activities.
SIM-E	SIM5	Music learning tasks involve designing, testing, and improving creative products or solutions.
SIM-E	SIM6	I have opportunities to explain the principles behind musical sounds, rhythms, structures, or digital effects.
SIM-E	SIM7	Group projects in music class require collaboration across different knowledge areas.
SIM-E	SIM8	Music learning activities allow me to transform abstract knowledge into creative musical expression.
SIM-E	SIM9	SIM-E activities give me space to explore multiple possible solutions rather than a single correct answer.
CSE	CSE1	I am confident that I can generate original ideas in music-related learning tasks.
CSE	CSE2	I can find creative ways to solve problems that arise during music or STEAM projects.
CSE	CSE3	I believe I can improve a creative product through repeated revision.
CSE	CSE4	I can combine different kinds of knowledge to produce novel outcomes.
CSE	CSE5	When a creative task is difficult, I still believe I can make progress.
CSE	CSE6	I am able to express my own ideas in creative project work.
LE	LE1^†^	I actively participate in SIM-E learning activities.
LE	LE2	I concentrate on tasks when working on interdisciplinary music projects.
LE	LE3	I complete assigned learning tasks carefully in SIM-E activities.
LE	LE4	I feel interested when music learning is connected with other disciplines.
LE	LE5^†^	I feel emotionally involved when creating or presenting SIM-E products.
LE	LE6	I enjoy collaborating with classmates during music-centered STEAM tasks.
LE	LE7	I try to understand the deeper ideas behind interdisciplinary music tasks.
LE	LE8^†^	I reflect on feedback and revise my work during SIM-E projects.
LE	LE9^†^	I look for additional information or strategies when I encounter difficulty in SIM-E learning.
IC	IC1	I can propose new ideas when facing learning or problem-solving tasks.
IC	IC2	I can combine different ideas to develop useful solutions.
IC	IC3	I can evaluate and improve my ideas after receiving feedback.
IC	IC4	I am willing to try unfamiliar methods when solving problems.
IC	IC5	I can transform creative ideas into concrete products, performances, or plans.
IC	IC6	I can collaborate with others to develop innovative outcomes.
IC	IC7	I can apply what I learn in one context to create solutions in another context.

SIM-E, STEAM-integrated music education; CSE, creative self-efficacy; LE, context-specific learning engagement; IC, innovative competence. ^†^Item wording explicitly references the focal SIM-E context.
